# Multidimensional digital biomarker phenotypes for mild cognitive impairment: considerations for early identification, diagnosis and monitoring

**DOI:** 10.3389/fdgth.2024.1265846

**Published:** 2024-03-06

**Authors:** Tracy Milner, Matthew R. G. Brown, Chelsea Jones, Ada W. S. Leung, Suzette Brémault-Phillips

**Affiliations:** ^1^ Faculty of Rehabilitation Medicine, University of Alberta, Edmonton, AB, Canada; ^2^ Department of Computing Science, University of Alberta, Edmonton, AB, Canada; ^3^ Heroes in Mind, Advocacy and Research Consortium (HiMARC), University of Alberta, Edmonton, AB, Canada; ^4^ Department of Occupational Therapy, Faculty of Rehabilitation Medicine, University of Alberta, Edmonton, AB, Canada; ^5^ Neuroscience and Mental Health Institute, University of Alberta, Edmonton, AB, Canada

**Keywords:** biomarkers, digital, cognitive, MCI, clinical, phenotypes, machine learning

## Abstract

Mild Cognitive Impairment (MCI) poses a challenge for a growing population worldwide. Early identification of risk for and diagnosis of MCI is critical to providing the right interventions at the right time. The paucity of reliable, valid, and scalable methods for predicting, diagnosing, and monitoring MCI with traditional biomarkers is noteworthy. Digital biomarkers hold new promise in understanding MCI. Identifying digital biomarkers specifically for MCI, however, is complex. The biomarker profile for MCI is expected to be multidimensional with multiple phenotypes based on different etiologies. Advanced methodological approaches, such as high-dimensional statistics and deep machine learning, will be needed to build these multidimensional digital biomarker profiles for MCI. Comparing patients to these MCI phenotypes in clinical practice can assist clinicians in better determining etiologies, some of which may be reversible, and developing more precise care plans. Key considerations in developing reliable multidimensional digital biomarker profiles specific to an MCI population are also explored.

## 1 Introduction

Mild Cognitive Impairment (MCI) poses a challenge for a growing population worldwide. Early identification of MCI is critical to providing the right interventions at the right time to maximize an individual's quality of life. Unlike conditions such as a bone fracture for which a definitive diagnosis can be made using imaging, it is more complex to determine whether decline in an individual's cognitive function is indicative of MCI. Predictive factors of increased future risk of experiencing MCI can also be difficult to isolate. Yet no gold standard currently exists to predict or identify MCI and traditional biomarkers such as imaging, genes or blood work have been used with mixed results (1, 2). A more reliable, valid, scalable and clinically useful method for predicting, diagnosing, and monitoring MCI is yet needed. Digital biomarkers captured through smart device technology widely used within the general population holds new promise.

MCI, while not a definitive diagnosis, represents an “in-between” condition between normal aging and early dementia (3, 4). A 2018 Alzheimer's Disease Facts and Figures, authored by the American Alzheimer's Association, anticipated that MCI will be diagnosed in 15%–20% of people older than 65 presenting an urgent need for earlier and more accurate identification (2, 5). MCI significantly lowers quality of life and, if it advances to dementia, eventually requires intense caregiving and support. The lifetime cost per individual with dementia is estimated to be USD$341,000. Earlier diagnosis can facilitate reversal or slowing of decline and improve or sustain quality of life longer. From a cost benefit perspective per individual, every year a person is diagnosed earlier translates to an estimated USD$30,000 savings, providing incentive to healthcare payers to invest in early identification and interventions (5).

MCI is currently diagnosed based on (a) an impairment in one or more cognitive domains, (b) the individual or their informant reporting decline, (c) preservation of daily functioning and (d) the criteria for dementia having not been met (3). Early recognition is important for identifying potentially reversible causes of MCI (4, 6), such as dehydration, vitamin deficiency, uncontrolled or underlying chronic disease like diabetes and cardiovascular disease, certain temporary and treatable infections, depression, chronic pain and more (7). Early recognition of MCI may involve different profiles related to various potential etiologies.

A wide range of risk factors are associated with MCI. In addition to cognition, these can include age, gender, education, diabetes or hypertension, genotypes, vitamin D deficiency, sleep-disordered breathing, sepsis, depression, and anxiety (8, 9). As such, when trying to identify early indications of MCI that reflect its multidimensional nature, genetic, medical history, demographic and psychosocial, physical and functional data must also be considered (4). Multiple features may need to be present together to create unique risk or diagnostic profiles. Without analysis of a comprehensive and multidimensional data set, key markers may be missed that can identify specific etiologies, provide precise diagnosis, or recommend the most effective treatment path (6, 7). A multidimensional approach to MCI that can stratify into different types is therefore needed. This may be beneficial to multidisciplinary clinical teams as they each formulate intervention recommendations.

## 2 Traditional approaches in MCI diagnosis

Traditional approaches for MCI diagnosis have included imaging and neuropsychological testing. Several challenges and limitations, however, are associated with this approach. Imaging has not been definitive, can be costly and is less accessible (10). There is no gold standard to follow in neuropsychological testing for MCI (2, 4, 10), and many people do not have access to or funding for such testing. Potentially long wait times can also obfuscate the benefits of early identification and the testing process itself can be long and arduous for the individual (10). Additionally, ceiling effects limit the effectiveness of traditional paper and pencil neuropsychological tests. As a result, milder cognitive dysfunction that may be present earlier can be missed by traditional tests. These tests also have difficulty predicting real world performance and are susceptible to reliability issues such as differences in test batteries and administration between assessors (6, 10, 11). While traditional paper and pencil neuropsychological tests can effectively compare individual to group performance on the same test, it becomes problematic when an individual's characteristics do not match those of the comparison group (e.g., due to comorbidities or use of certain medications) (4). These tests can also be less sensitive to assessing clinically significant changes in performance over time (10). Adding further complexity to diagnosis of MCI are potentially overlooked psychosocial or physical dimensions that can present in addition to cognitive changes (4).

The Montreal Cognitive Assessment (MOCA) is an example of a screening tool often used to make a diagnosis of MCI. A score below 26 (or in some settings below 23) of a possible 30 points suggests MCI (4). An individual scoring below this cut-off would demonstrate an inability to complete tasks like counting or spelling backwards, copying a cube, or immediately repeating a set of words. This level of cognitive performance is already an indication of more severe cognitive dysfunction, such that the individual was likely already demonstrating milder cognitive dysfunction in complex thinking activities (e.g., prospective memory, executive function) that were not identified earlier because they are not well-assessed using traditional tests (4).

In response to these challenges, clinicians have needed to take a more eclectic approach to formulating a differential clinical diagnosis and intervention strategy. This can include a combination of history taking, patient and family interviews, neuropsychological testing, and functional observation (8). Initial assessment results may not reveal the etiology of the MCI without additional testing or longitudinal monitoring, which makes the process more clinically and time intensive. Additional training, experience and skill are often required, reducing accessibility to care by limiting the number of clinicians who can assess and treat for MCI (8, 9).

## 3 The promise of digital biomarkers

Digital biomarkers have the potential to assist in the early identification of MCI and improve the accuracy and efficiency of the initial clinical assessment. Collected and measured by means of digital devices, digital biomarkers are objective, quantifiable, physiological and behavioral data (12). While traditional biomarkers for MCI can be based on genes, molecules, cells, or neural data, digital biomarkers can be derived from these as well as from physiological factors (e.g., heart rate, speech), cognitive factors (e.g., memory, divided attention, information processing speed), behavioral factors (e.g., geolocation data, screen use, typing), social factors (e.g., event attendance, call logs, app engagement), digital footprint analysis (e.g., electronic health records) and patient and family reports (e.g., daily function, sleep, nutrition) (13). This broad definition of digital biomarkers that is not limited to only correlated digital versions of traditional biomarkers has been criticized for diverging too far from a measurement taken directly in the body (14). Others, however, have advocated for collaboration with regulatory bodies to define different types (e.g., actively/passively acquired, point-in-time/continuous) or classes (e.g., direct/indirect, existing/novel) of digital biomarkers that could enable corresponding validation pathways (15). We prefer the broader definition of digital biomarkers. It can better represent the multidimensional nature of MCI and improve clinical utility by highlighting possible areas for intervention that a narrower definition may exclude.

Digital biomarkers for MCI can have several purposes. These can include: (a) risk evaluation, (b) prediction, (c) diagnosis, (d) prognostics, and (e) monitoring (16–18). Taking a composite approach that can combine traditional and digital biomarkers can assist with all purposes (4, 6, 19). Given the multiple MCI etiologies, each often with different presenting features, it is probable that there will be different digital biomarker profiles or phenotypes that represent various etiologies. For example, for MCI related specifically to dementia, different types can include Alzheimer's, Vascular, Frontotemporal Dementia, Parkinson's Disease Dementia and more, each with their own distinguishing presenting features. This is similar to psychiatry where the creation of digital phenotypes gathered from behavior, cognition and mood has been highlighted as promising for a measurement-based approach to patient care (20). Applying such an approach to MCI also holds much promise.

## 4 Validating multidimensional phenotypes for MCI

Validating biomarkers for MCI, whether traditional or digital, is a significant undertaking and more complex with novel biomarkers or biomarker phenotypes. To define a common nomenclature for biomarkers and biomarker validation, the Food and Drug Administration and the National Institutes of Health formed a joint task committee to develop an online, dynamic resource called the BEST (Biomarkers, EndpointS, and other Tools) (16). This resource outlines the process in selecting a biomarker for validation from evidentiary assessment, to utilizing it to collect data, analyzing it to determine if it is linked to the condition of interest and purpose, and verifying its algorithm.

Algorithmic gold standards for identifying MCI or predicting the transition between MCI and dementia are to date lacking (4). As a result, potential new digital biomarkers do not have existing traditional reference biomarkers against which they can be validated (17). Digital biomarkers currently showing promise include geolocation, keyboard interaction, and voice and speech analysis (20). The approach in these studies, which is an approach outlined in the BEST (14), is to develop a hypothesis on a biomarker to select for validation based on review of the evidence correlating it to MCI. A study that correlated human-computer interactions to traditional neuropsychological constructs is an example (21). This approach, however, can risk missing the identification of promising biomarkers as the study of digital biomarkers for MCI is relatively new and the evidence limited (8). Correlating to measures that have been found to lack sensitivity for MCI detection is another risk. This approach also does not lend itself to creating the multidimensional phenotypes likely needed for more sensitive and reliable detection and clinical utility. In clinical practice, assessment informs treatment planning. Assessments that rely on a limited number of biomarkers of MCI may not produce sufficient information to the clinician to determine etiologies (20) or understand what to modify or treat to reduce an individual's risk of MCI, reverse the MCI, or slow decline (8). More advanced approaches such as high-dimensional statistics and machine learning will be required to identify and validate multidimensional phenotypes.

The multidimensional nature of MCI itself poses unique challenges to biomarker validation. Thousands of potential digital biomarkers can be collected about one individual related to their cognition, behavior, physical health, psychosocial functioning and/or activities; this becomes exponential when collected longitudinally (6, 22). Further, because it is unlikely that one digital biomarker alone will predict risk, diagnosis, outcome, prognosis, or signify clinical change (2), diverse, rich, and large data sets will need to be analyzed. Advanced analytical approaches such as high-dimensional statistics and machine learning offer alternate approaches to the single variable hypothesis-based limitations described above and are essential for analyzing large, longitudinal data sets that can derive a constellation of digital biomarkers that serve as a phenotype for different types of MCI (6, 22, 23). This approach can be used to evaluate larger sets of potential digital biomarkers for associations with MCI to select the digital biomarkers with the most potential for validation.

When used with high-dimensional data, machine learning typically requires very large data sets that can be difficult to obtain or take significant time and cost to build (6, 24). This is particularly true of deep learning approaches with large models, which have demonstrated recent successes in other areas (e.g., ChatGPT). Limitations in the size of previously available datasets have imposed limitations on previous analyses using advanced statistics or machine learning. There are now, however, several large longitudinal brain health studies collecting more multi-dimensional data. The Canadian Longitudinal Study on Aging, for example, is collecting interviews, neuropsychological, questionnaires and physical data on 50,000 people for 20 years (25), Other examples include the UCSF Brain Health Registry (26), Ontario Brain Institute's BRAIN-CODE (27), the Alzheimer's Disease Neuroimaging Initiative (28) and more. Multi-modal studies leveraging deep machine learning are examples of research trending towards this multi-dimensional phenotype approach for MCI; some with several types of brain imaging and others combining demographic, neuropsychological and genetic data (29–33). These studies and datasets offer opportunities to employ machine learning, including deep learning, and aggregate existing datasets from multiple sources to create a rich, diverse, and comprehensive data set that can better represent the variety of types of biomarkers that should be considered for MCI. Collecting more digital footprint or mobile sensing type data could enrich the dataset for phenotype analysis.

Once digital biomarkers are successfully selected, the next challenge is to determine whether the selected biomarker validates for its intended purpose (e.g., risk, diagnosis, prediction) or if the biomarker might be a confounding variable that is moderating or modifying another more direct relationship (18, 19). With digital biomarkers for the purpose of monitoring, there is an additional challenge in identifying what thresholds, “cut-offs” or degree of change in a digital biomarker is required to support changes in care pathways or clinical decision making. The level of significance for digital biomarker change can be difficult to ascertain, especially at the level of the individual (11, 17). Teams with multi-disciplinary expertise, such as researchers, clinicians, statisticians, data scientists, computational neuroscientists, and technology manufacturers, will be needed to address the complexities and maximize the potential for success in identifying MCI digital biomarker phenotypes (18).

## 5 Additional considerations

Identifying multidimensional digital biomarker phenotypes for MCI, regardless of the purpose of the digital biomarker being pursued, poses unique measurement and validation challenges. In addition to the usual biases, confounding variables and other common measurement issues in study design, the research team attempting to validate digital biomarkers for MCI must also consider several factors. These include: (1) data standardization, integrity and verifiability, (2) ethics and consent, and (3) generalizability and accessibility specific to an MCI population.

### 5.1 Data standardization, integrity and verifiability

A biomarker's ability to collect data reliably and accurately is foundational to its validation (18, 34). The data must be collected in a standardized manner each time and include a process to verify that the data belongs to the person to whom it is attributed. This can be particularly challenging when the data is gathered remotely or passively or if the digital device itself, the method of collection or the software algorithm changes over time (19). It is important to understand whether the context in which the data was collected is impacting its reliability or accuracy, such as environmental distractions or individual factors such as the person's effort and how they were feeling at the time of collection (e.g., pain, fatigue, anxiety) (11, 35, 36). In addition, because individuals with MCI can experience memory issues, they may be poor historians in self-reported data (35). Collateral verification may be needed (i.e., from health records, health providers, or family/friends) to ensure data integrity (11). Another consideration relates to verifiability of the collection of digital biomarkers that use proprietary software and whether the algorithms or collection methods can be disclosed to the research team for verification or any restrictions or conditions from the intellectual property holder(s) will limit publications (18, 19). Flaws in these areas can lead to unreliable or inaccurate data that when analyzed can lead to flawed conclusions.

### 5.2 Ethics and consent

Ethical use of personal health data including its de-identification process and compliance with relevant privacy and security regulations must be verified (6, 18–20). When data collection is occurring passively through programs that monitor app use or how someone is using a digital device, it is important to ascertain whether explicit individual consent has been given for the purpose of health research (18, 20). Whether a person who in fact does have MCI can make a fully informed decision and provide consent to use of their data is a further consideration (6).

### 5.3 Generalizability and accessibility

Those who use digital devices are a subset of the MCI population and therefore generalizability of the digital biomarker MCI phenotypes to those who do not use technology can be limited (13). Even within this subset, some will not want to participate in research (13) or others may not want to be assessed for MCI for a variety of reasons (7). This introduces sampling bias and limits generalizability (5, 13). Some health providers may be reluctant to promote study participation if they believe early identification of MCI can cause more anxiety and distress to patients than the benefits of early intervention (7, 12). An individual's accessibility to MCI assessment can also be limited by the type of technology required (18), internet access, insurance reimbursement or payment coverage, or access to healthcare or research (6, 36).

## 6 Discussion

Most individuals have a digital health footprint that they create through their interactions with the healthcare system, digital applications, and digital devices in general. The analysis of this comprehensive digital health footprint to identify multidimensional digital biomarker phenotypes for MCI offers new promise and opportunities for MCI identification and clinical decision support tools.

Validation, however, is complex and challenging. There are no gold standard biomarkers that can serve as reference biomarkers for validating new biomarkers for MCI. A review of the validation process corresponding to different types of digital biomarkers is needed. Advanced methods, such as high-dimensional statistics and deep machine learning methods will be required. Large, longitudinal datasets can be further enriched by including more multi-dimensional data sources (e.g., electronic medical records, digital footprint, mobile sensing). A multidisciplinary approach is best positioned to achieve successful outcomes. Complementary perspectives can help capture a broad range of factors that need to be considered when analyzing digital biomarkers across multiple domains. A methodical approach to study design and analysis would need to be taken by any research team to evaluate data source comprehensiveness, integrity and generalizability, and consider issues specific to the MCI population. The team would also need to document how challenges were addressed and acknowledge limitations on study conclusions for challenges that could not be anticipated or managed.

Early identification of MCI and timely initiation of therapies and treatments can positively impact the increasing number of people experiencing cognitive decline. There is a need for MCI assessment and clinical decision support tools that are sensitive, reliable, efficient, standardized, accessible to more health providers, and that provide sufficient information from which to develop personalized and precise treatment plans. Comparing an individual's health profile to validated multidimensional MCI digital biomarker phenotypes can directly assist health providers to identify their patient's risk factors, monitor their profile over time for changes, diagnose MCI with etiology stratification, predict the likelihood of progression into dementia or another condition, and recommend the interventions to which their patient may best respond. Early recognition of factors that are reversible or modifiable to reduce risk or slow progression can produce optimal health outcomes.

The possibility of developing multidimensional phenotypes of digital biomarkers capable of reliably identifying MCI is compelling, despite associated challenges, and offers emerging opportunities. The potential to contribute to enhanced quality of life for those experiencing MCI, as well as health system and socioeconomic benefits, is significant. The use of digital biomarkers for early identification of MCI is poised to help make everyday life better for a growing number of people experiencing MCI and those who support them.

## Data Availability

The original contributions presented in the study are included in the article/Supplementary Material, further inquiries can be directed to the corresponding author.

## References

[1] Tang F Uchendu I Wang F Dodge HH Zhou J. Scalable diagnostic screening of mild cognitive impairment using AI dialogue agent. Sci Rep. (2020) 10(1):1–11. 10.1038/s41598-020-61994-0 32235884 PMC7109153

[2] Chehrehnegar N Nejati V Shati M Rashedi V Lotfi M Adelirad F Early detection of cognitive disturbances in mild cognitive impairment: a systematic review of observational studies. Psychogeriatrics. (2020) 20(2):212–28. 10.1111/psyg.12484 31808989

[3] Sanford AM. Mild cognitive impairment. Clin Geriatr Med. (2017) 33(3):325–37. 10.1016/j.cger.2017.02.005 28689566

[4] Cavedoni S Chirico A Pedroli E Cipresso P Riva G. Digital biomarkers for the early detection of mild cognitive impairment: artificial intelligence meets virtual reality. Front Hum Neurosci. (2020) 14:245. 10.3389/fnhum.2020.00245 32848660 PMC7396670

[5] *2018 Alzheimer’s disease facts and figures Includes a Special report on the financial and personal benefits of early diagnosis*. Available online at: https://www.alz.org/media/HomeOffice/Facts%20and%20Figures/facts-and-figures.pdf (accessed April 19, 2022).

[6] Graham SA Lee EE Jeste DV van Patten R Twamley EW Nebeker C Artificial intelligence approaches to predicting and detecting cognitive decline in older adults: a conceptual review. Psychiatry Res. (2020) 284:112732. 10.1016/j.psychres.2019.112732 31978628 PMC7081667

[7] Sabbagh MN Boada M Borson S Chilukuri M Doraiswamy PM Dubois B Rationale for early diagnosis of mild cognitive impairment (MCI) supported by emerging digital technologies. J Prev Alzheimers Dis. (2020) 7(3):158–64. 10.14283/jpad.2020.19 32463068

[8] Langa KM Levine DA. The diagnosis and management of mild cognitive impairment. JAMA. (2014) 312(23):2551. 10.1001/jama.2014.13806 25514304 PMC4269302

[9] Jongsiriyanyong S Limpawattana P. Mild cognitive impairment in clinical practice: a review article. Am J Alzheimers Dis Other Demen. (2018) 33(8):500–7. 10.1177/1533317518791401 30068225 PMC10852498

[10] Gielis K vanden Abeele ME de Croon R Dierick P Ferreira-Brito F van Assche L Dissecting digital card games to yield digital biomarkers for the assessment of mild cognitive impairment: methodological approach and exploratory study. JMIR Serious Games. (2021) 9(4):e18359. 10.2196/18359 34734825 PMC8603181

[11] Kolanowski A Gilmore-Bykovskyi A Hill N Massimo L Mogle J. Measurement challenges in research with individuals with cognitive impairment. Res Gerontol Nurs. (2019) 12(1):7–15. 10.3928/19404921-20181212-06 30653646 PMC6500490

[12] Piau A Wild K Mattek N Kaye J. Current state of digital biomarker technologies for real-life, home-based monitoring of cognitive function for mild cognitive impairment to mild Alzheimer disease and implications for clinical care: systematic review. J Med Internet Res. (2019) 21(8):e12785. 10.2196/12785 31471958 PMC6743264

[13] Torous J Rodriguez J Powell A. The new digital divide for digital biomarkers. Digit Biomark. (2017) 1(1):87–91. 10.1159/000477382 29104959 PMC5665386

[14] Montag C Elhai JD Dagum P. On blurry boundaries when defining digital biomarkers: how much biology needs to be in a digital biomarker? Front Psychiatry. (2021) 12:740292. 10.3389/fpsyt.2021.740292 34658973 PMC8514660

[15] Au R Kolachalama VB Paschalidis ICH. Redefining and validating digital biomarkers as fluid, dynamic multi-dimensional digital signal patterns. Front Digit Health. (2022) 3:751629. 10.3389/fdgth.2021.751629 35146485 PMC8822623

[16] FDA-NIH Biomarker Working Group—Best (Biomarkers, Endpoints, and Other Tools). Silver Spring, MD: Food and Drug Administration (US) (2016). Available online at: https://www.ncbi.nlm.nih.gov/books/NBK338449/#top (accessed April 16, 2022). 27010052

[17] Califf RM. Biomarker definitions and their applications. Exp Biol Med (Maywood). (2018) 243(3):213–21. 10.1177/1535370217750088 29405771 PMC5813875

[18] Coravos A Goldsack JC Karlin DR Nebeker C Perakslis E Zimmerman N Digital medicine: a primer on measurement. Digit Biomark. (2019) 3(2):31–71. 10.1159/000500413 32095767 PMC7015383

[19] Babrak LM Menetski J Rebhan M Nisato G Zinggeler M Brasier N Traditional and digital biomarkers: two worlds apart? Digit Biomark. (2019) 3(2):92–102. 10.1159/000502000 32095769 PMC7015353

[20] Insel TR. Digital phenotyping: technology for a new science of behavior. JAMA. (2017) 318(13):1215–6. 10.1001/jama.2017.11295 28973224

[21] Dagum P. Digital biomarkers of cognitive function. NPJ Digit Med. (2018) 1(1):10. 10.1038/s41746-018-0018-4 31304295 PMC6550173

[22] Fisher CK Smith AM Walsh JR Simon AJ Edgar C Jack CR Machine learning for comprehensive forecasting of Alzheimer’s disease progression. Sci Rep. (2019) 9:13622. 10.1038/s41598-019-49656-2 31541187 PMC6754403

[23] Wiens J Shenoy ES. Machine learning for healthcare: on the verge of a major shift in healthcare epidemiology. Clin Infect Dis. (2017) 66(1):149–53. 10.1093/cid/cix731 PMC5850539 29020316

[24] Zhang D Shen D. Semi-supervised multimodal classification of Alzheimer’s disease. 2011 IEEE International Symposium on Biomedical Imaging: From Nano to Macro [Internet]. (2011). p. 1628–31. Available online at: http://resolver.scholarsportal.info/resolve/19457928/v2011inone/1628_smcoad.xml (accessed February 2, 2024).

[25] Canadian Longitudinal Study on Aging. *Canadian longitudinal study on aging*. Available online at: https://www.clsa-elcv.ca/ (accessed May 6, 2023).

[26] UCSF Brain Health Registry. Available online at: https://www.brainhealthregistry.org/ (accessed May 6, 2023).

[27] OBI. Brain-CODE. *Ontario Brain Institute*. (2024). Available online at: https://braininstitute.ca/research-data-sharing/brain-code (accessed February 2, 2024).

[28] ADNI|Access data. Available online at: https://adni.loni.usc.edu/data-samples/access-data/ (accessed February 2, 2024).

[29] Jiao Z Chen S Shi H Xu J. Multi-modal feature selection with feature correlation and feature structure fusion for MCI and AD classification. Brain Sci. (2022) 12(1):80. 10.3390/brainsci12010080 35053823 PMC8773824

[30] Xiong J Zhu H Li X Hao S Zhang Y Wang Z Auto-classification of Parkinson’s disease with different motor subtypes using arterial spin labelling MRI based on machine learning. Brain Sci. (2023) 13(11):1524–4. 10.3390/brainsci13111524 38002484 PMC10670033

[31] Spasov S Passamonti L Duggento A Liò P Toschi N. A parameter-efficient deep learning approach to predict conversion from mild cognitive impairment to Alzheimer’s disease. NeuroImage. (2019) 189:276–87. 10.1016/j.neuroimage.2019.01.031 30654174

[32] Katabathula S Davis PB Xu R. Comorbidity-driven multi-modal subtype analysis in mild cognitive impairment of Alzheimer’s disease. Alzheimer’s & Dement. (2022) 19(4):1428–39. 10.1002/alz.12792 PMC10040466 36166485

[33] Long Z Li J Fan J Li B Du Y Qiu S Identifying Alzheimer’s disease and mild cognitive impairment with atlas-based multi-modal metrics. Front Aging Neurosci. (2023) 15:1212275. 10.3389/fnagi.2023.1212275 37719872 PMC10501142

[34] Aronson JK Ferner RE. Biomarkers-a general review. Curr Protoc Pharmacol. (2017) 76:9.23.1–7. 10.1002/cpph.19 28306150

[35] Far MS Eickhoff SB Goni M Dukart J. Exploring test-retest reliability and longitudinal stability of digital biomarkers for Parkinson disease in the m-power data set: cohort study. J Med Internet Res. (2021) 23(9):e26608. 10.2196/26608 34515645 PMC8477293

[36] Sabbagh MN Boada M Borson S Doraiswamy PM Dubois B Ingram J Early detection of mild cognitive impairment (MCI) in an at-home setting. J Prev Alzheimers Dis. (2020) 7(3):171–8. 10.14283/jpad.2020.22 32463070

